# Mitochondrial DNA copy number variation across three generations: a possible biomarker for assessing perinatal outcomes

**DOI:** 10.1186/s40246-023-00567-4

**Published:** 2023-12-15

**Authors:** Hisanori Fukunaga, Atsuko Ikeda

**Affiliations:** 1 Center for Environmental and Health Sciences, Hokkaido University, N12 W7 Kita-ku, Sapporo, 060-0812 Japan; 2 Faculty of Health Sciences, Hokkaido University, Sapporo, Japan

**Keywords:** Birth cohort, Cord blood, DNA copy number variation, Maternal exposure, Mitochondrial DNA

## Abstract

**Background:**

Mitochondria have their own circular multi-copy genome (mtDNA), and abnormalities in the copy number are implicated in mitochondrial dysfunction, which contributes to a variety of aging-related pathologies. However, not much is known about the genetic correlation of mtDNA copy number across multiple generations and its physiological significance.

**Methods:**

We measured the mtDNA copy number in cord blood or peripheral blood from 149 three-generation families, specifically the newborns, parents, and grandparents, of 149 families, totaling 1041 individuals. All of the biological specimens and information were provided by the Tohoku Medical Megabank Project in Japan. We also analyzed their maternal factors during pregnancy and neonatal outcomes.

**Results:**

While the maternal peripheral blood mtDNA copy number was lower than that of other adult family members, it was negatively correlated with cord blood mtDNA copy number in male infants. Also, cord blood mtDNA copy numbers were negatively correlated with perinatal outcomes, such as gestation age, birth weight, and umbilical cord length, for both male and female neonates. Furthermore, the mtDNA copy number in the infants born to mothers who took folic acid supplements during pregnancy would be lower than in the infants born to mothers who did not take them.

**Conclusions:**

This data-driven study offers the most comprehensive view to date on the genetic and physiological significance of mtDNA copy number in cord blood or peripheral blood taken from three generations, totaling more than 1000 individuals. Our findings indicate that mtDNA copy number would be one of the transgenerational biomarkers for assessing perinatal outcomes, as well as that appropriate medical interventions could improve the outcomes via quantitative changes in mtDNA.

**Supplementary Information:**

The online version contains supplementary material available at 10.1186/s40246-023-00567-4.

## Background

Eukaryotic cells, which arose about two billion years ago through the symbiosis of archaea and aerobic bacteria, acquired mitochondria because they represented an efficient energy-producing system. The mitochondrial DNA (mtDNA) codes for the central genes of the mitochondrial energy–generating process of oxidative phosphorylation, and mutations in the mtDNA can cause many of the symptoms associated with common metabolic and degenerative diseases [1]. Human mtDNA is maternally inherited and comprises a cyclic multicopy genome containing 16,569 base pairs, with usually tens to thousands of copies per cell [2].

Previous studies have indicated that peripheral blood mtDNA copy numbers decrease with age and are negatively associated with age-related events, such as all-cause mortality [3, 4]. Recently, the European Prospective Investigation into Cancer and Nutrition (EPIC) cohort study indicated that mtDNA copy numbers from blood samples are associated with an increased risk of developing estrogen receptor-positive breast cancer [5]. The German Chronic Kidney Disease (GCKD) study, a prospective national CKD cohort study, showed that a low mtDNA copy number is related to mortality in patients with CKD [6]. A low copy number is also correlated with age-related pathological conditions, such as cardiovascular diseases [7] and cognitive dysfunction [8].

The developmental origins of health and disease (DOHaD) theory [9–15], which states that foetal exposomes shape disease predisposition and influence the risk of morbidity in adulthood, has been supported by numerous epidemiological studies and animal experiments, following the pioneering studies on low-birthweight (LBW) infants by David Barker and his colleagues in the 1980s [16–18]. An epidemiological study from 2006 reported that newborns with abnormal birth weights have reduced mtDNA content, when compared to newborns of appropriate weight [19]. Based on further accumulating evidence, abnormalities in mtDNA copy numbers would be considered one of the presumed links between abnormal foetal growth and metabolic and cardiovascular complications in later life.

In the present study, we comprehensively examined mtDNA copy numbers based on cord and peripheral blood from 149 three-generation families, consisting of newborns, mothers, fathers, maternal grandmothers, maternal grandfathers, paternal grandmothers, and paternal grandfathers, for a total of 1041 individuals. In addition, from the point of view of the DOHaD theory, we determined the correlation between maternal factors during pregnancy and neonatal outcomes. The purpose of this data-driven study is to determine the significance of mtDNA copy number as a biomarker. All of the biological specimens and information were provided by the Tohoku Medical Megabank (TMM) Project [20, 21].

## Results

### Distribution of mtDNA copy number from cord blood and peripheral blood

**Fig. 1:**
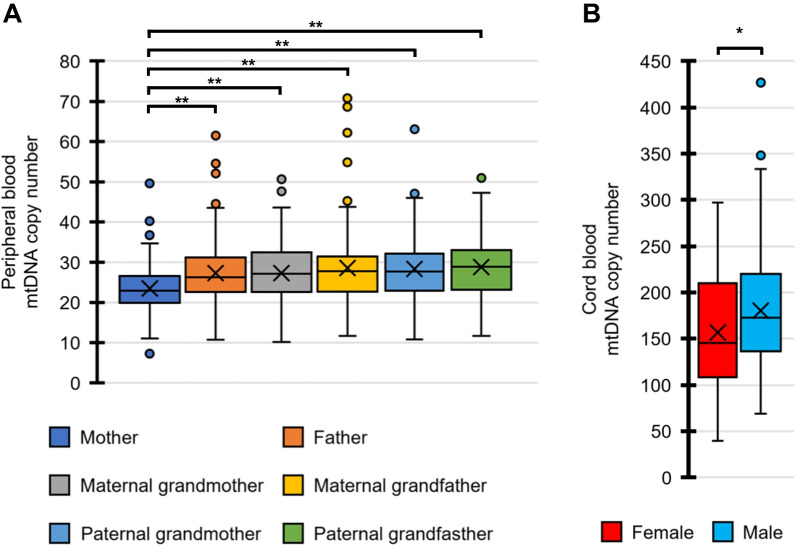
Distribution of mtDNA copy number from peripheral blood and cord blood. **A** Box-and-whisker plots show the distribution of peripheral blood mtDNA copy numbers in the mother (n = 149), father (n = 149), maternal grandmother (n = 148), maternal grandfather (n = 147), paternal grandmother (n = 149), and paternal grandfather (n = 149) groups. The Dunnett test showed that mothers’ numbers (control) are significantly lower than those of the other groups. **B** Box-and-whisker plots show the distribution of cord blood mtDNA copy numbers in the female (n = 68) and male newborn groups (n = 82). The Mann–Whitney U test showed that female newborns’ numbers are significantly lower than those of male newborns. Statistically significant differences were found when *p < 0.05 and **p < 0.01

As shown in Fig. 1A, the peripheral blood mtDNA copy numbers of the mothers (n = 149), fathers (n = 149), maternal grandmothers (n = 148), maternal grandfathers (n = 147), paternal grandmothers (n = 149), and paternal grandfathers (n = 149) were determined. The mtDNA copy numbers were significantly lower in the mothers than in the other family members (*p* < 0.01, Dunnett’s test). In this study, there is no correlation between the age and peripheral blood mtDNA copy numbers for each position in the family (see Additional file 1: Fig. S1). The cord blood mtDNA copy numbers of the female (n = 68) and male newborns (n = 82, including male twins) were also determined (Fig. 1B). The mtDNA copy numbers in the female newborns were significantly lower than those of males (*p* = 0.047, Mann–Whitney test).

### Correlation of mtDNA copy number between parents and children

**Fig. 2:**
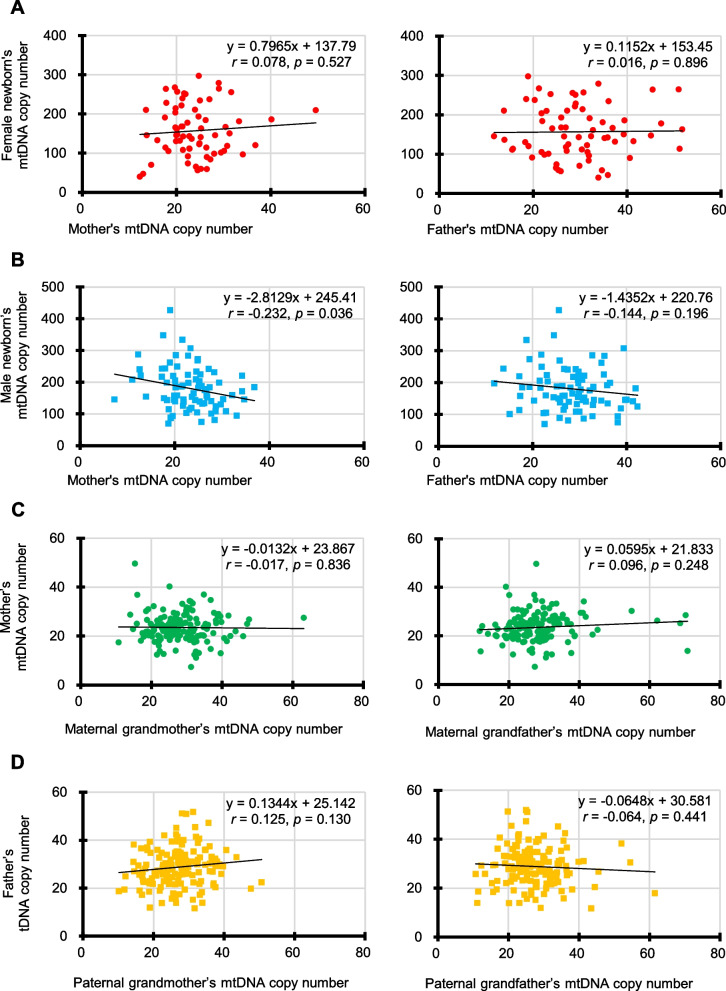
Correlation of mtDNA copy number between parents and children. **A** Scatterplots show the Pearson’s correlation coefficients of mtDNA copy number between the female newborn and mother groups (left) and father groups (right). **B** Scatterplots show the Pearson’s correlation coefficients of mtDNA copy number between the male newborn and mother groups (left) and father groups (right). **C** Scatterplots show the Pearson’s correlation coefficients of mtDNA copy number between the mother and maternal grandmother groups (left) and maternal grandfather groups (right). **D** Scatterplots show the Pearson’s correlation coefficients of mtDNA copy number between the father and paternal grandmother groups (left) and paternal grandfather groups (right). Because four outcome measures (the mtDNA copy numbers for female newborns, male newborns, mothers, and fathers) were tested against two hypothesized predictors (the mtDNA copy numbers for their mothers and their fathers), a Bonferroni-adjusted significance level of 0.00625 (= 0.05/8)

Figure 2 shows the Pearson’s correlation coefficients for mtDNA copy numbers between female newborns and mothers, female newborns and fathers, male newborns and mothers, male newborns and fathers, mothers and maternal grandmothers, mothers and maternal grandfathers, fathers and paternal grandmothers, and fathers and paternal grandfathers. Interestingly, there was a weak negative correlation between the male newborns and mothers (*r* = − 0.232, *p* = 0.036), although this genetic correlation did not reach the Bonferroni-adjusted statistical significance level at *p* = 0.00625. Significant correlations were not found in the other parent–child relationships, including fathers and paternal grandmothers. In addition, no significant correlation was observed between newborns and grandparents.

### Maternal characteristics during pregnancy and neonatal outcomes

Table 1 shows maternal characteristics and neonatal outcomes based on comprehensive data questionnaires (e.g., regarding exercise, smoking, and alcohol consumption), haematological test results, and neonatal information derived from the TMM Project. The mean age of the mothers at enrolment was 32.1, with those aged 35 and over accounting for 31.5% of the total. The mothers’ mean pre-pregnancy body mass index (BMI) was 21.89 kg/m^2^, and 9.4% of participants had values of less than 18.50. Their mean haemoglobin (HGB) level in early pregnancy was 12.5 g/dl, and 10.7% of participants had values of less than 11.5. Further, 66.4% of the mothers engaged in folic acid (FA) supplementation during pregnancy. There were no current smokers among the mothers, but 22.8% were former smokers, and 39.6% had been second-hand smoking in the past year. Although 21.1% of mothers had a history of fertility treatments, just a few pregnant women had a history of hypertension (2.0%) or diabetes (2.0%).

**Table 1:** Characteristics of mothers and newborns

Characteristics	Mean ± SD or n (%)
Maternal characteristics	
Maternal age (years)	32.1 ± 4.2
Advanced maternal age (≥ 35)	47 (31.5)
Pre-pregnancy BMI (kg/m^2^)	21.89 ± 3.73
Lean (< 18.5)	14 (9.4)
Hemoglobin (g/dl)	12.5 ± 0.9
Anemia (< 11.5)	16 (10.7)
Folic acid supplementation during pregnancy (Yes)	99 (66.4)
Exercise habit during pregnancy (Yes)	77 (51.7)
Ex-smoking (Yes)	34 (22.8)
Passive smoking in the past 1 year (Yes)	59 (39.6)
History of fertility care (Yes)	21 (14.1)
History of hypertension (Yes)	3 (2.0)
History of diabetes (Yes)	3 (2.0)
Neonatal outcomes	
Sex, male	82 (54.7)
Gestational age (weeks)	39.2 ± 1.4
Birth weight (g)	3058.9 ± 400.7
Low birth weight (< 2500)	13 (8.7)
Height (cm)	49.6 ± 2.2
Head circumference (cm)	33.4 ± 1.5
Chest circumference (cm)	31.8 ± 1.7
Placental weight (g)	552.7 ± 93.2
Umbilical cord length (cm)	55.5 ± 10.9
Umbilical cord artery blood pH	7.31 ± 0.08

Continuous variables are presented by mean ± SD (normally distributed)

Categorial variables are expressed by n (%)

Males accounted for 54.7% of newborns, outnumbering females. In this study, we found a significant sex difference in head circumference (*p* = 0.005). Also, there were possible differences in gestational age (*p* = 0.038) and placental weight (*p* = 0.042), although they did not reach the Bonferroni-adjusted statistical significance level at *p* = 0.01 (Additional file 1: Fig. S2). The LBW infants (< 2500 g) accounted for 8.7% of newborns. The mtDNA copy numbers of normal-birthweight newborns were significantly lower than those of LBW infants (*p* < 0.001, Mann–Whitney test) (Additional file 1: Fig. S3).

### Correlation among maternal characteristics, neonatal outcomes, and mtDNA copy number

The Pearson’s correlation coefficients suggested a possible positive correlation between only the mtDNA copy number and HGB for male newborns’ mothers (*r* = 0.221, *p* = 0.046), although this correlation did not reach the Bonferroni-adjusted statistical significance level at *p* = 0.00417 (Additional file 1: Fig. S4). As shown in Additional file 1: Fig. S5, the mtDNA copy numbers of male infants born to mothers under the age of 35 were smaller than those of males born to mothers aged 35 and over (*p* = 0.032, Mann–Whitney test). The mtDNA copy numbers of females’ mothers with BMIs under 18.50 were also smaller than those of female infants’ mothers with pre-pregnancy BMIs of 18.50 or over (*p* = 0.015). However, these differences did not reach the adjusted statistical significance level at *p* = 0.00417.

**Fig. 3:**
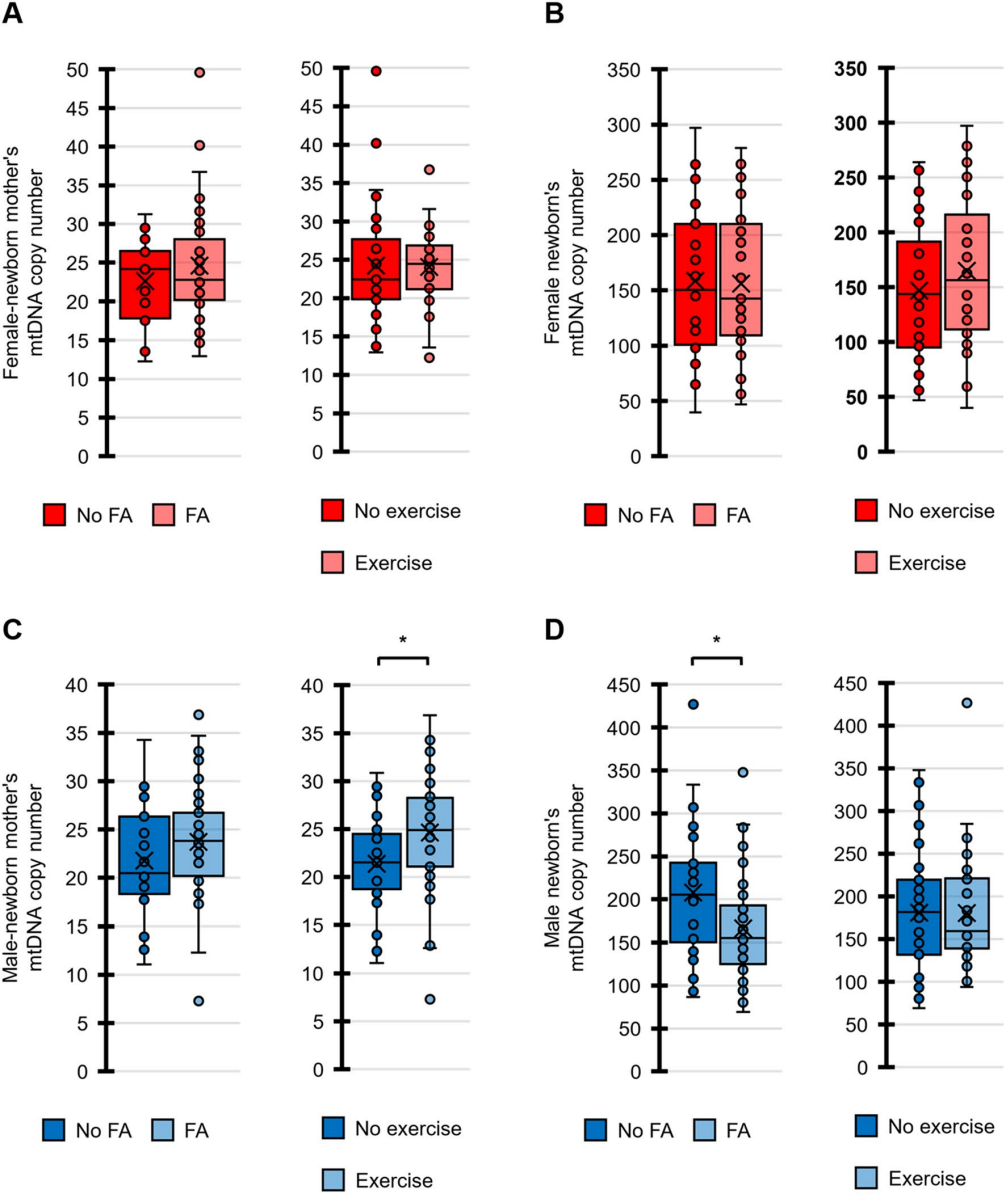
Comparison of mtDNA copy number for maternal lifestyle factors. **A** Comparison of female-newborn mothers’ mtDNA copy numbers based on engaging in FA supplementation (left) and their exercising or not (right). **B** Comparison of female newborns’ mtDNA copy numbers based on engaging in FA supplementation (left) and their exercising or not (right). **C** Comparison of male-newborn mothers’ mtDNA copy numbers based on their engaging in FA supplementation (left) and their exercising or not (right). **D** Comparison of male newborns’ mtDNA copy numbers based on their engaging in FA supplementation (left) and their exercising or not (right). Because four outcome measures were tested against two hypothesized predictors, a Bonferroni-adjusted significance level of 0.00625 (= 0.05/8) was calculated. *Statistically significant difference

Figure 3 shows that the mtDNA copy numbers of male-newborn mothers who did not exercise during pregnancy were significantly lower than those of males’ mothers who exercised (*p* < 0.001, Mann–Whitney test). In addition, the mtDNA copy numbers were smaller for the male newborns with FA supplementation during pregnancy than without the supplementation (*p* = 0.0065), although the difference did not reach the adjusted statistical significance level at *p* = 0.00625.

**Fig. 4:**
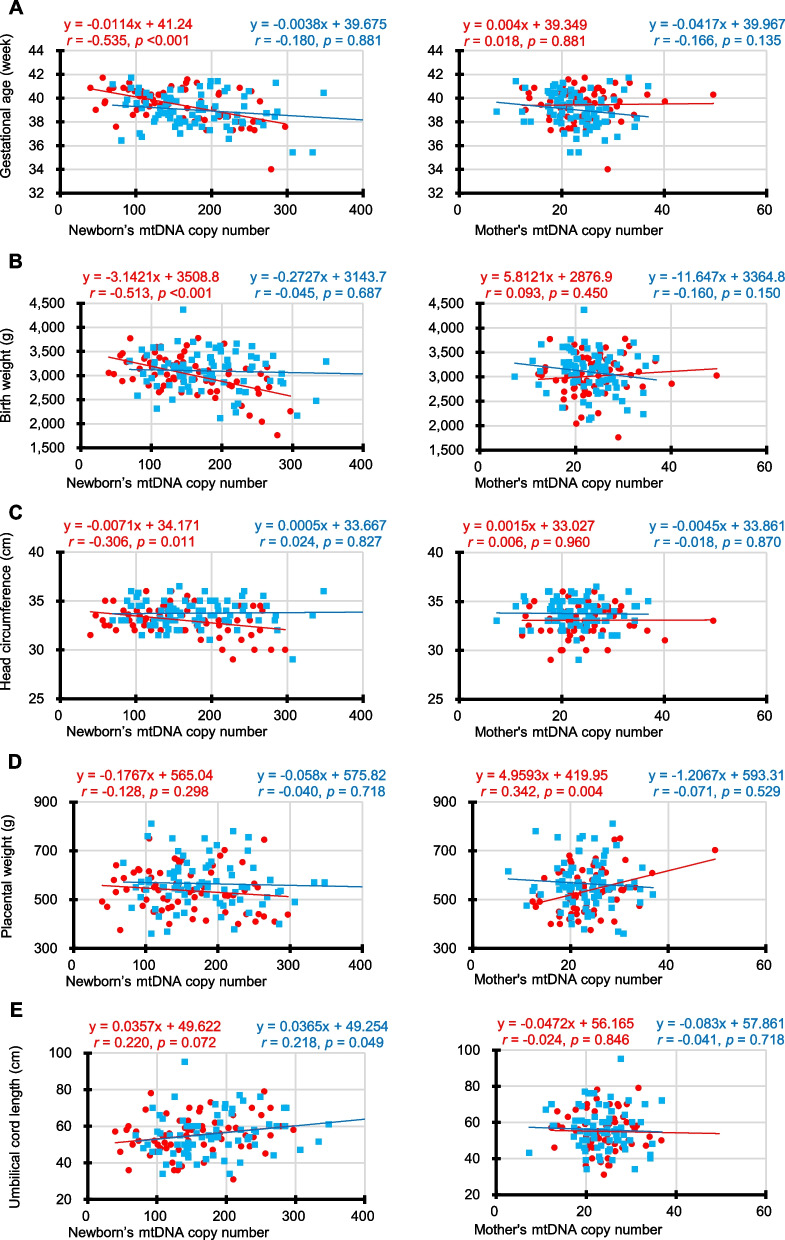
Correlation between neonatal outcomes and mtDNA copy numbers. **A** Scatterplots show the Pearson’s correlation coefficients between the gestational age (week) and mtDNA copy numbers of newborns (left) and mothers (right). **B** Scatterplots show the correlations between the birth weight (g) and mtDNA copy numbers of newborns (left) and mothers (right). **C** Scatterplots show the correlations between the head circumference (cm) and mtDNA copy numbers of newborns (left) and mothers (right). **D** Scatterplots show the correlations between the placental weight (g) and mtDNA copy numbers of newborns (left) and mothers (right). **E** Scatterplots show the correlations between the umbilical cord length (cm) and mtDNA copy numbers of newborns (left) and mothers (right). Female and male newborns’ data are shown in red and blue, respectively. Because five outcome measures were tested against two hypothesized predictors, a Bonferroni-adjusted significance level of 0.005 (= 0.05/10) was calculated

As shown in Fig. 4, the Pearson’s correlation coefficients showed that the female newborns’ mtDNA copy numbers were significantly correlated with gestational age (*r* = − 0.535, *p* < 0.001) and birth weight (*r* = − 0.153, *p* < 0.001). In addition, they were negatively correlated head circumference (*r* = − 0.306, *p* = 0.011), although it did not reach the adjusted statistical significance level at *p* = 0.005. However, no significant correlation was found between the female newborns’ mtDNA copy numbers and the SD scores for birth weight and head circumference (Additional file 1: Fig. S6). Interestingly, the mtDNA copy numbers of female-newborn mothers were significantly correlated with placental weight (*r* = 0.342, *p* = 0.004).

### Independent factors associated with mtDNA copy number

To determine the significance of mtDNA copy number as a perinatal biomarker, as shown in Table 2, a variable selection multiple regression analysis using the stepwise backward elimination method was performed. This examined the correlations among maternal characteristics, neonatal outcomes, and mtDNA copy numbers.

**Table 2:** Independent variables in association with mothers’ and newborns’ mtDNA copy numbers

Variables	Reference	All newborn-mother				Male newborn-mother				Female newborn-mother			
		β	95% CI		*p*-value	β	95% CI		*p*-value	β	95% CI		*p*-value
Mother’s mtDNA copy number													
Maternal characteristics													
Maternal age (years)	≥ 35 vs < 35 years	–				–				–			
Pre-pregnancy BMI	< 18.5 vs ≥ 18.5 kg/m^2^	− 3.73	− 7.13	− 0.34	0.031	–				− 4.58	− 9.95	0.79	0.093
Haemoglobin	< 11.5 vs ≥ 11.5 g/dl	–				− 3.59	− 6.91	− 0.28	0.034	–			
Folic acid supplementation during pregnancy	No vs Yes	1.82	− 0.21	3.85	0.078	–				–			
Exercise habit during pregnancy	No vs Yes	1.75	− 0.21	3.70	0.079	2.59	0.17	5.00	0.036	–			
Ex-smoking	No vs Yes	–				–				–			
Passive smoking in the past 1 year	No vs Yes	–				–				–			
History of fertility care	No vs Yes	–				–				–			
Neonatal outcomes													
Sex	Male vs female	–				–				–			
Gestational age	week (continuous)	–				–				–			
Birth weight	< 2500 vs ≥ 2500 g	–				–				–			
Height	cm (continuous)	–				–				–			
Head circumference	cm (continuous)	–				–				–			
Placental weight	g (continuous)	–				–				0.02	0.00	0.03	0.036
Umbilical cord length	cm (continuous)	–				–				–			
Newborn’s mtDNA copy number													
Maternal characteristics													
Maternal mtDNA copy number	Continuous	–				− 3.26	− 5.99	− 0.53	0.020	–			
Maternal age	≥ 35 vs < 35 years	–				–				–			
Pre-pregnancy BMI	< 18.5 vs ≥ 18.5 kg/m^2^	–				–				–			
Haemoglobin	< 11.5 vs ≥ 11.5 g/dl	–				–				–			
Folic acid supplementation during pregnancy	No vs Yes	− 26.36	− 49.51	− 3.22	0.026	− 31.47	− 61.64	− 1.30	0.041	–			
Exercise habit during pregnancy	No vs Yes	–				–				–			
Ex-smoking	No vs Yes	–				–				–			
Passive smoking in the past 1 year	No vs Yes	–				–				–			
History of fertility care	No vs Yes	–				–				–			
Neonatal outcomes													
Sex	Male vs female	–				–				–			
Gestational age	week (continuous)	− 16.92	− 25.30	− 8.53	< 0.001	− 14.01	− 26.38	− 1.64	0.027	− 17.67	− 29.59	− 5.74	0.004
Birth weight	< 2500 vs ≥ 2500 g	70.25	26.80	113.70	0.002	63.62	3.77	123.48	0.038	67.56	9.13	126.00	0.024
Height	cm (continuous)	–				–				–			
Head circumference	cm (continuous)	8.82	0.84	16.80	0.031	12.20	− 0.28	24.68	0.055	−			
Placental weight	g (continuous)	–				–				–			
Umbilical cord length	cm (continuous)	1.94	1.01	2.87	< 0.001	2.22	0.81	3.63	0.002	1.26	0.02	2.51	0.047

β (95%CI) were calculated by stepwise backward elimination linear regression analysis to determine the independent variables in association with mtDNA copy numbers

All variables of maternal characteristics or infant characteristics were introduced into each model at same time. “–”suggests the variable eliminated from the final model

Because six sets of the multiple regression analysis were performed for the mtDNA copy numbers of mothers and newborns for all newborn-mother, male newborn-mother, and female newborn-mother pairs, a Bonferroni-adjusted significance level of 0.00833 (= 0.05/6) was calculated

The objective variables were maternal and neonatal mtDNA copy numbers. Maternal age at enrolment, pre-pregnancy BMI, HGB in early pregnancy, FA supplementation during pregnancy, exercise habits during pregnancy, smoking history, second-hand smoking history in the past year, and infertility treatment history were selected from among the maternal characteristics as explanatory variables. The maternal mtDNA copy numbers were used as an explanatory variable for the neonatal mtDNA copy numbers. Sex, gestational age (week), birthweight (g), height (cm), head circumference (cm), placental weight (g), and umbilical cord length (cm) were also selected from among the neonatal outcomes.

**Fig. 5:**
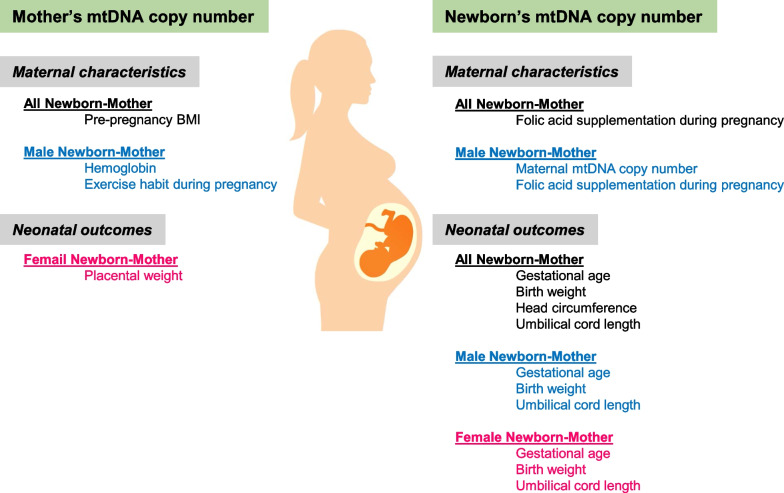
Summary of independent variables in association with mothers’ and newborns’ mtDNA copy numbers

According to the results, the significant variables for maternal mtDNA copy numbers were pre-pregnancy BMI (*p* = 0.031); for male newborns’ maternal mtDNA copy numbers, HGB (*p* = 0.034) and exercise habits were significant (*p* = 0.036); for female infants’ maternal mtDNA copy numbers, only placental weight was significant (*p* = 0.036). By contrast, the significant variables for all newborns’ mtDNA copy numbers were FA supplementation (*p* = 0.026), gestation age (*p* < 0.001), birthweight (*p* = 0.002), head circumference (*p* = 0.031), and umbilical cord length (*p* < 0.001); for male newborns’ mtDNA copy numbers, maternal mtDNA copy numbers (*p* = 0.020), FA supplementation (*p* = 0.041), gestational age (*p* = 0.027), birthweight (*p* = 0.038), and umbilical cord length (*p* = 0.002) were significant; for female infants’ mtDNA copy numbers, gestation age (*p* = 0.004), birthweight (*p* = 0.024), and umbilical cord length (*p* = 0.047) were significant. Figure 5 shows all these findings.

A point that should be noted is that, if the Bonfferoni correction is applied to these results to avoid false positives (type-I errors), for all newborns’ mtDNA copy numbers, gestation age, birth weight, and umbilical cord length; for male infants’ mtDNA copy numbers, only umbilical cord length; and for female infants’ mtDNA copy numbers, only gestational age remained statistically significant.

## Discussion

To our knowledge, this is the first study to comprehensively determine cord or peripheral blood mtDNA copy numbers in more than 1000 individuals from three-generation families to elucidate the genetic correlates of mtDNA copy numbers. In addition, as the TMM Project progresses, we expect to find that a correlation between adulthood outcomes and newborns’ and mothers’ mtDNA copy numbers will be uncovered. Our data-driven study revealed the following three significant results regarding mtDNA copy number.

First, we found a possible correlation between the mtDNA copy numbers of cord blood from male infants and the mtDNA copy numbers of their mother’s peripheral blood (*p* = 0.036). This suggests a genetic relationship in terms of mtDNA copy numbers between parents and offspring, and provides novel insights into the inheritance of mtDNA copy numbers. However, this correlation did not reach the Bonferroni-adjusted statistical significance level at *p* = 0.00625. Thus, further investigation is needed to confirm this finding in future. Also, this correlation may be specific to the perinatal period, because no correlation was found for the peripheral blood mtDNA copy numbers between fathers and paternal grandmothers.

Secondly, we found that the mtDNA copy numbers of newborns and their mothers during the perinatal period were significantly correlated with neonatal outcomes. However, no causal relationship can immediately be discerned from the correlations found in this study. Nevertheless, according to recent epidemiological studies, maternal exposure to various environmental factors during pregnancy is significantly associated with an elevation in mtDNA copy numbers in cord blood [2]. By contrast, in the present study, the cord blood mtDNA copy numbers were negatively correlated with gestational age, and increase low birth weight for both male and female neonates. Taken together, the changes in cord blood mtDNA copy numbers due to exposure to environmental stresses may serve as a biomarker for assessing perinatal outcomes.

Finally, we found a possible relationship between newborns and mothers who took FA supplements during pregnancy in terms of their mtDNA copy numbers (*p* = 0.026), suggesting that maternal FA intake may reduce the mtDNA copy number in cord blood. This indicates that some neonatal outcomes can be modified by adequate interventions for mothers during pregnancy that result in quantitative changes in mtDNA. Supplementation with FA, a synthetic form of folate, is well known to cause a significant reduction in the risk of neural tube defects (NTD) [22]. In fact, strong evidence has been provided for a role for the mitochondrial folate pathway in neural tube development in an *MTHFD1L*-knockout mouse model, which presents NTD with 100% penetrance even when fed a folate-proficient diet [23]. Several previous studies have also demonstrated the importance of folate in maintaining mitochondrial function [24]. Our findings suggest that FA supplementation during pregnancy has the potential to prevent NTD while also improving other birth outcomes through its effects on mitochondrial function. However, no significant correlation between the mtDNA copy number and the FA supplementation during pregnancy was observed in the multiple regression analysis adjusted by Bonferroni correction. Further research is needed to confirm this finding, and may have important implications for perinatal care and prevention.

This study had several technical limitations. First, although available biospecimens were collected from over 1000 individuals, the sample size of each group remained small. Even with proper statistical analysis, we cannot deny the possibility of a type-II error in the results. Furthermore, Bonferroni correction controls for false positives (type-I errors) become very conservative as the number of tests increases. This can also increase the risk of generating false negatives (type-II two errors). Second, the target population of this study was not very diverse in terms of genetic background because of the limited area from which the participants were recruited. Third, some participant bias may have been present because the participants were cooperating in a cohort study at one of two academic research institutions. In fact, the proportion of LBW infants was lower than the proportion indicated by recent trends in Japan overall [25], suggesting the possibility of bias toward the healthy population. In addition, the study design did not rigorously control for blood composition, which could be a substantial confounder. A further limitation was the lack of an independent validation cohort in which the findings were replicated.

## Conclusions

We present mitochondrial DNA copy numbers from 149 three-generation families, totaling 1,041 individuals, and evaluate the correlation with birth outcomes. The result suggested a possible negative correlation of maternal mtDNA copy number in peripheral blood collected during pregnancy with the cord blood mtDNA copy number of the male infants. The cord blood mtDNA copy numbers were also negatively correlated with gestational age, birth weight, and umbilical cord length for both male and female neonates. In addition, a possible relationship was detected between the mtDNA copy numbers of male newborns and mothers who took FA supplements during pregnancy, indicating that neonatal outcomes can be modified by adequate interventions for mothers during pregnancy via quantitative changes in mtDNA. Further investigation is required to validate these findings, and may shed light on the emerging role of mtDNA copy number as a transgenerational biomarker.

## Methods

### Information and biological samples from the TMM project

We used the data and biospecimens from the Tohoku Medical Megabank Project Birth and Three-Generation Cohort Study (TMM BirThree Cohort Study) [26], which has been operated by Tohoku University’s Tohoku Medical Megabank Organization (ToMMo) and Iwate Medical University’s Iwate Tohoku Medical Megabank Organization (IMM) since 2012. In fact, they have provided an analytical platform that researchers and health professionals are able to perform collaborative studies by using genome and medical data [27, 28]. All the participants were recruited in the Tohoku region (in northeastern Japan) between 2013 and 2017. The participants in the TMM Project are still under follow-up and expected to provide further epidemiological data on growth; biological aging; and the incidence of various diseases, including lifestyle-related diseases [29].

The comprehensive data obtained from questionnaires, hematological test results, neonatal information, and the DNA samples extracted from buffy coats of cord blood or peripheral blood were provided to the present study. In principle, the blood samples were collected from the participants at the time they were enrolled in the TMM BirThree Cohort Study. In particular, the mothers' peripheral blood was collected during their pregnancies.

### Determination of mtDNA copy number

To determine the mtDNA copy numbers from the DNA samples, with the Applied Biosystems’ StepOne real-time PCR analysis (Thermo Fisher Scientific Inc., MA, USA), we used the Human Mitochondrial DNA Monitoring Primer Set (Cat. #7246) and TB Green^®^ Premix Ex Taq™ II (Tli RNase H Plus), 5 × 5 mL, ROX Plus (Cat. # RR82WR) (Takara BIO Inc, Shiga, Japan). The thermal profile was as follows: 2 min at 98 °C, 40 cycles at 98 °C for 10 s, 15 s at 60 °C, and 30 s at 68 °C. To measure mtDNA copy numbers relative to nuclear DNA (nDNA) [30], the primer set contained two primer pairs each for detecting mtDNA and nDNA, for a total of four primers. These primers target two genes on mtDNA (*ND1*, *ND5*) and two genes on nDNA (*SLCO2B1*, *SERPINA*). To determine the mtDNA copy number, first, the difference in Ct values of the *ND1*/*SLCO2B1* pair was measured and then ΔCt1 (Ct value of *SLCO2B1*–Ct value of *ND1*) was calculated. Similarly, the difference in Ct values for the *ND5*/*SERPINA1* pair was measured and ΔCt2 (Ct value of *SERPINA1*–Ct value of *ND5*) was calculated. Finally, 2N (N = ΔCt) was obtained from the ΔCt1 and ΔCt2 values, and the average of these two values was used as the copy number.

### Statistics

The Dunnett’s test was used to compare the mtDNA copy numbers of the mothers (control) with those of the other family members (Fig. 1A). The Mann–Whitney test was employed for comparisons between the two groups (Figs. 1B and 3; Additional file 1: Figs. S2, S3 and S5). The Pearson’s correlation coefficient was also employed to evaluate the correlations between the two groups (Figs. 2 and 4; Additional file 1: Figs. S1, S4 and S6). Multiple regression analysis with stepwise backward elimination method was used to predict independent variables associated with mtDNA copy number from maternal factors during pregnancy and neonatal outcomes (Table 2). Statistical significance was set at *p* < 0.05 both side for a continuous model. In addition, to account for the increased possibility of type-I errors due to multiple testing, we used Bonferroni correction to adjust the significance level. We used the Statistical Package for the Social Sciences (SPSS) ver. 27J (IBM, NY, USA).

### Supplementary Information

**Additional file 1**. **Figure S1**: Correlation between age and mtDNA copy number. **Figure S2**: Comparison of female and male newborns for birth outcomes. **Figure S3**: Comparison of newborns’ and mothers’ mtDNA copy numbers. **Figure S4**: Correlation between maternal physical factors and mtDNA copy number. **Figure S5**: Comparison of mtDNA copy number for maternal physical factors. **Figure S6**: Correlation between neonatal outcome SD scores and mtDNA copy numbers.

## Data Availability

All data are available in the main text or the supplementary materials.

## Abbreviations

BMI: Body mass index
DOHaD: Developmental origins of health and disease
EPIC: European Prospective Investigation into Cancer and Nutrition
FA: Folic acid
GCKD: German Chronic Kidney Disease
HGB: Haemoglobin
IMM: Iwate Medical University’s Iwate Tohoku Medical Megabank Organization
LBW: Low-birthweight
mDNA: Mitochondrial DNA
NTD: Neural tube defects
TFAM: The mitochondrial transcription factor A
TMM BirThree Cohort Study: Tohoku Medical Megabank Project Birth and Three-Generation Cohort Study
ToMMo: Tohoku Medical Megabank Organization
