# Supplementary material for: Mitochondrial DNA copy number variation across three generations: a possible biomarker for assessing perinatal outcomes
Source: Hum Genomics. 2023 Dec 15;17:113. doi: 10.1186/s40246-023-00567-4 (PMC10722810; doi:10.1186/s40246-023-00567-4)
Supplement: Supplementary file 1 — Additional file 1. Figure S1: Correlation between age and mtDNA copy number. Figure S2: Comparison of female and male newborns for birth outcomes. Figure S3: Comparison of newborns’ and mothers’ mtDNA copy numbers. Figure S4: Correlation between maternal physical factors and mtDNA copy number. Figure S5: Comparison of mtDNA copy number for maternal physical factors. Figure S6: Correlation between neonatal outcome SD scores and mtDNA copy numbers. [file 40246_2023_567_MOESM1_ESM.pdf]

Supplementary materials for

“Mitochondrial DNA copy number variation across three generations: A possible biomarker for assessing perinatal outcomes”

Hisanori Fukunaga and Atsuko Ikeda

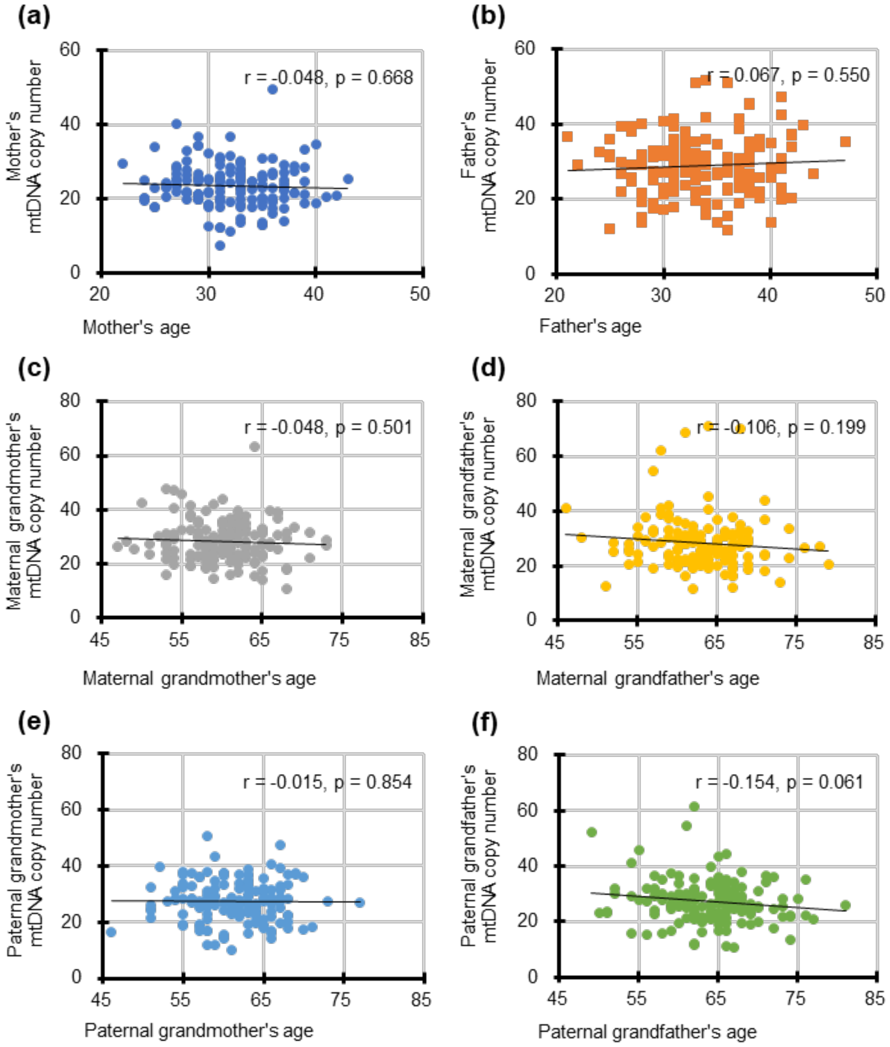

Supplemental Figure S1. Correlation between age and mtDNA copy number.

(**a-f**) Scatterplots show Pearson’s correlation coefficients between age at enrolment and mtDNA copy numbers of the mother (**a**), father (**b**), maternal grandmother (**c**), maternal grandfather (**d**), paternal grandmother (**e**), and paternal grandfather groups (**f**).

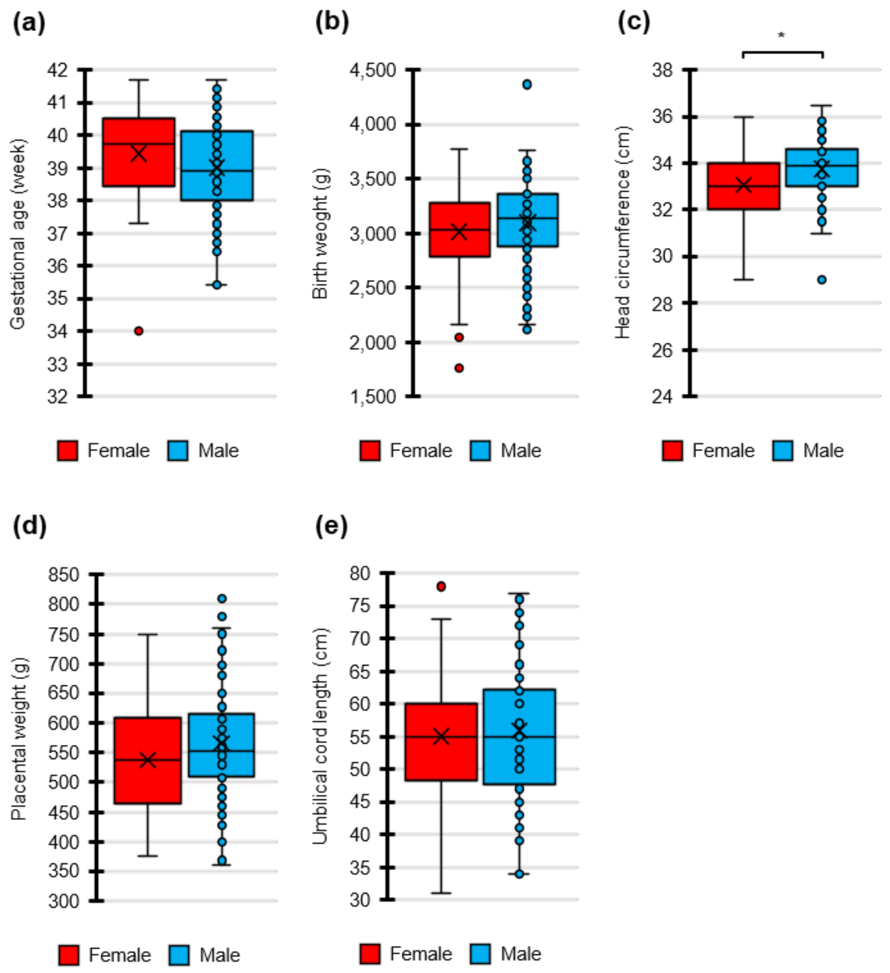

Supplemental Figure S2. Comparison of female and male newborns for birth outcomes.

(**a-e**) Comparisons of female and male newborns' gestational age (**a**), birth weight (**b**), head circumference (**c**), placental weight (**d**), and umbilical cord length (**e**). A Bonferroni-adjusted significance level of 0.01 (=0.05/5) was calculated. *Statistically significant difference.

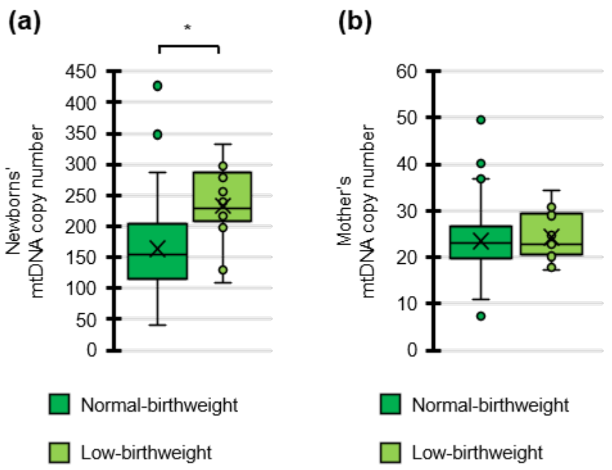

Supplemental Figure S3. Comparison of newborns' and mothers' mtDNA copy numbers.

(**a**) Comparison of newborns' mtDNA copy numbers based on birth weight (g), either under 2500 (Low-birthweight, LBW) or 2500 and over (Normal-birthweight, NBW). (**b**) Comparison of mothers' mtDNA copy numbers based on birth weight, either LBW or NBW. A Bonferroni-adjusted significance level of 0.25 (=0.05/2) was calculated. *Statistically significant difference.

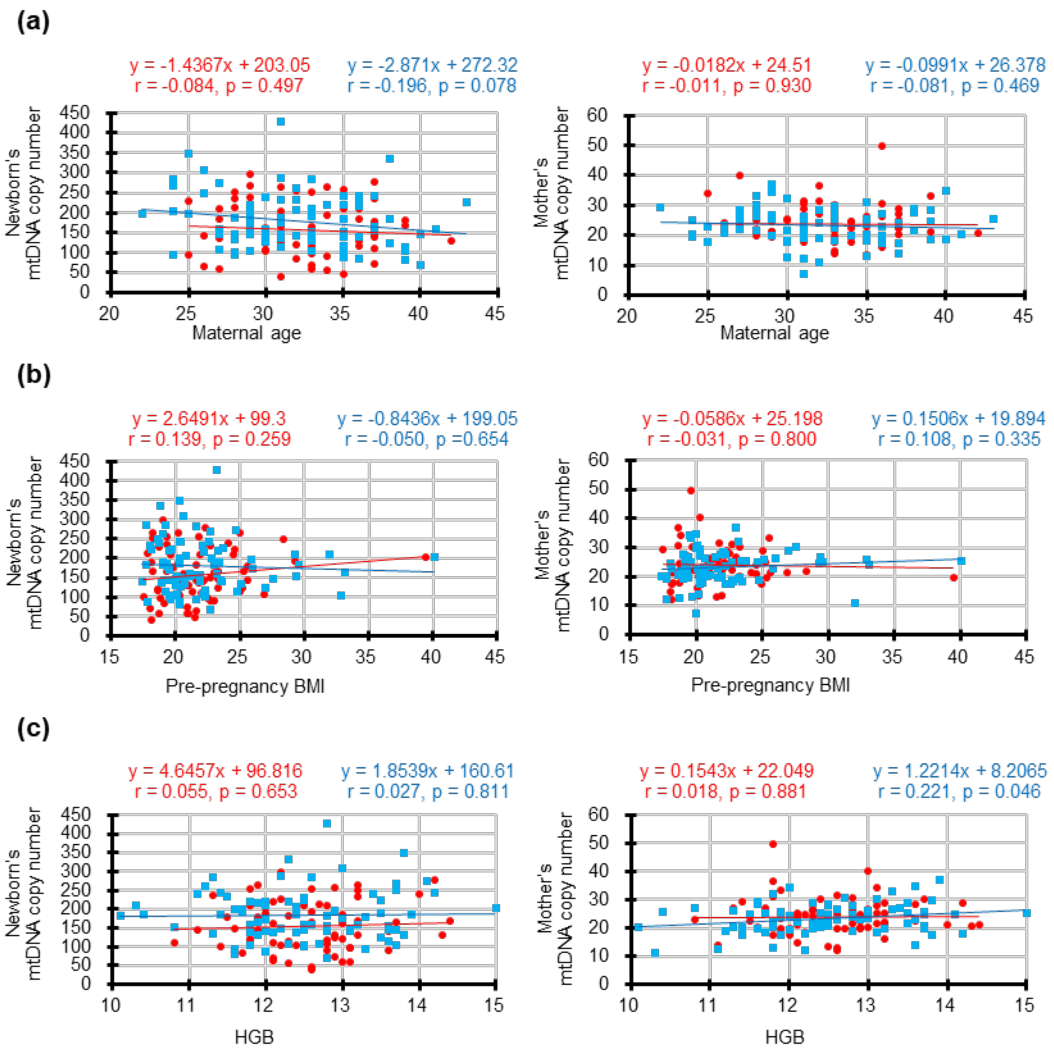

Supplemental Figure S4. Correlation between maternal physical factors and mtDNA copy number.

(**a**) Scatterplots show Pearson's correlation coefficients between maternal age at enrollment and mtDNA copy numbers of newborns (left) and mothers (right). (**b**) Scatterplots show Pearson's correlation coefficients between the pre-pregnancy BMI and mtDNA copy numbers of newborns (left) and mothers (right). (**c**) Scatterplots show Pearson's correlation coefficients between the HGB and mtDNA copy numbers of newborns (left) and mothers (right). Data for mothers of female and male newborns are shown in red and blue, respectively. A Bonferroni-adjusted significance level of 0.00417 (= 0.05/12) was calculated.

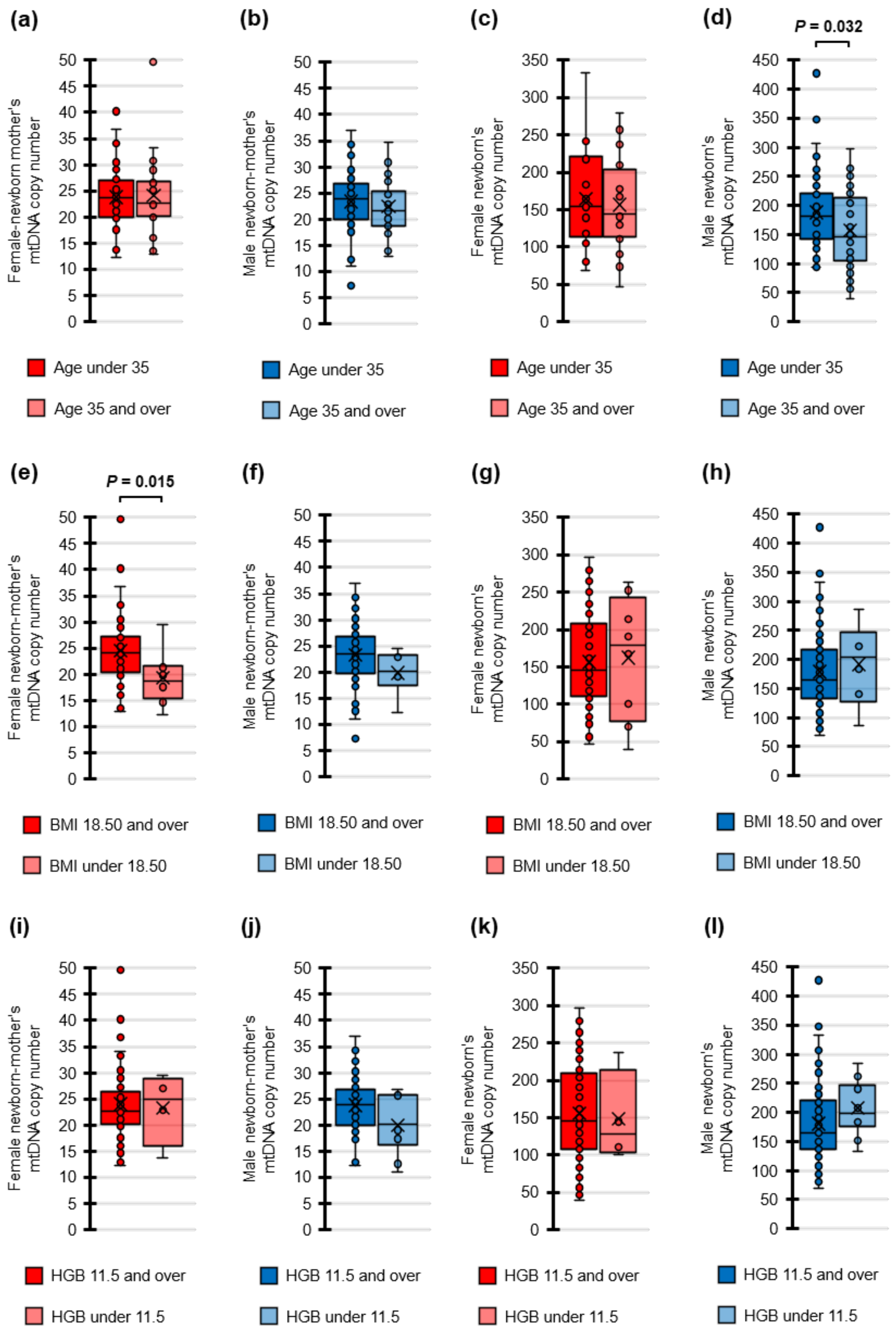

Supplemental Figure S5. Comparison of mtDNA copy number for maternal physical factors.

(**a**) Comparison of female-newborn mothers' mtDNA copy numbers based on their age, either under 35 years or 35 and over. (**b**) Comparison of male-newborn mothers' mtDNA copy numbers based on their age, either under 35 years or 35 and over. (**c**) Comparison of female newborns' mtDNA copy numbers based on their mothers' age, either under 35 years or 35 and over. (**d**) Comparison of male newborns' mtDNA copy numbers based on their mothers' age, either under 35 years or 35 and over.

(**e**) Comparison of female-newborn mothers' mtDNA copy numbers based on their BMI, either 18.50 and over or under 18.50. (**f**) Comparison of male-newborn mothers' mtDNA copy numbers based on their BMI, either 18.50 and over or under 18.50. (**g**) Comparison of female newborns' mtDNA copy numbers based on their mothers' BMI, either 18.50 and over or under 18.50. (**h**) Comparison of male newborns' mtDNA copy numbers based on their mothers' BMI, either at 18.50 and over or under 18.50. (**i**) Comparison of female-newborn mothers' mtDNA copy numbers based on their HGB, either 11.5 and over or under 11.5. (**j**) Comparison of male-newborn mothers' mtDNA copy numbers based on their HGB, either 11.5 and over or under 11.5. (**k**) Comparison of female newborns' mtDNA copy numbers based on their mothers' HGB, either 11.5 and over or under 11.5. (**l**) Comparison of male newborns' mtDNA copy numbers based on their mothers' HGB, either 11.5 and over or under 11.5. Data for mothers of female and male newborns are shown in red and blue, respectively. A Bonferroni-adjusted significance level of 0.00417 (= 0.05/12) was calculated.

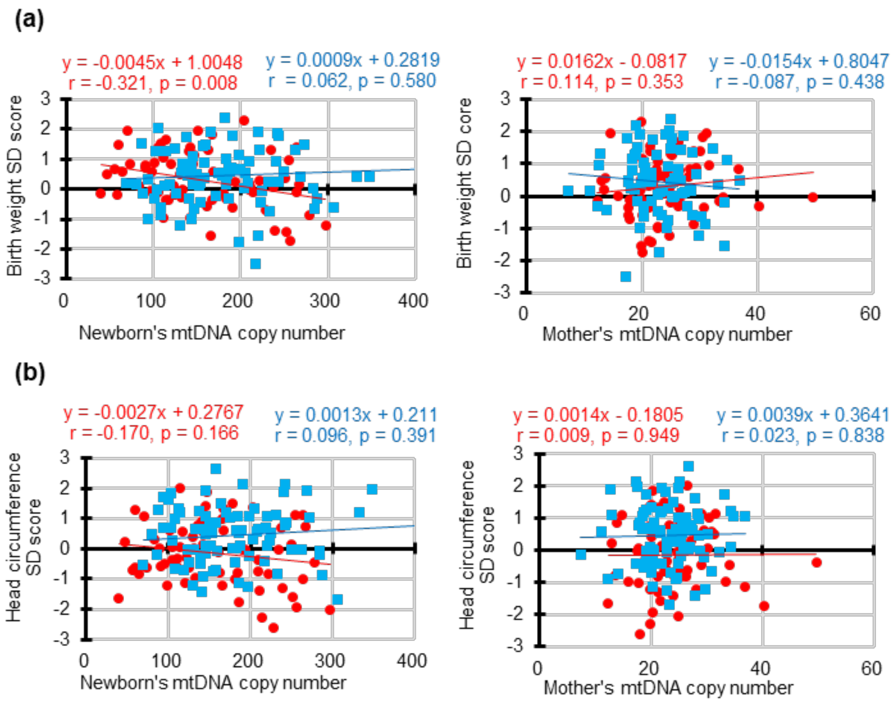

Supplemental Figure S6. Correlation between neonatal outcome SD scores and mtDNA copy numbers.

(**a**) Scatterplots show Pearson's correlation coefficients between birthweight SD score and mtDNA copy numbers of newborns (left) and mothers (right). (**b**)Scatterplots show the correlations between the head circumference SD score and mtDNA copy numbers of newborns (left) and mothers (right). Data for mothers of female and male newborns are shown in red and blue, respectively. A Bonferroni-adjusted significance level of 0.00625 (=0.05/8) was calculated.

* The Japanese Association for Human Auxology, the Japanese Society for Pediatric Endocrinology, and the Japan Society for Neonatal Health and Development have collaborated to create a Microsoft Excel file that allows the calculation of standardized anthropometric values at birth by gestational age for Japanese infants (available at http://jspe.umin.jp/medical/keisan.html). In the present study, this file was used to calculate SD scores for weight and head circumference.
